# Halide Mixing in Cs_2_AgBi(I_*x*_Br_1–*x*_)_6_ Double Perovskites: A Pathway to Tunable Excitonic Properties

**DOI:** 10.1021/acs.jpcc.4c04453

**Published:** 2024-08-26

**Authors:** Raisa-Ioana Biega, Huygen J. Jöbsis, Zamorano Gijsberg, Maxim Hüskens, Eline M. Hutter, Linn Leppert

**Affiliations:** † MESA+ Institute for Nanotechnology, University of Twente, 7500 AE Enschede, The Netherlands; ‡ Debye Institute for Nanomaterials Science, Utrecht University, Princetonlaan 8, 3584 CB Utrecht, The Netherlands

## Abstract

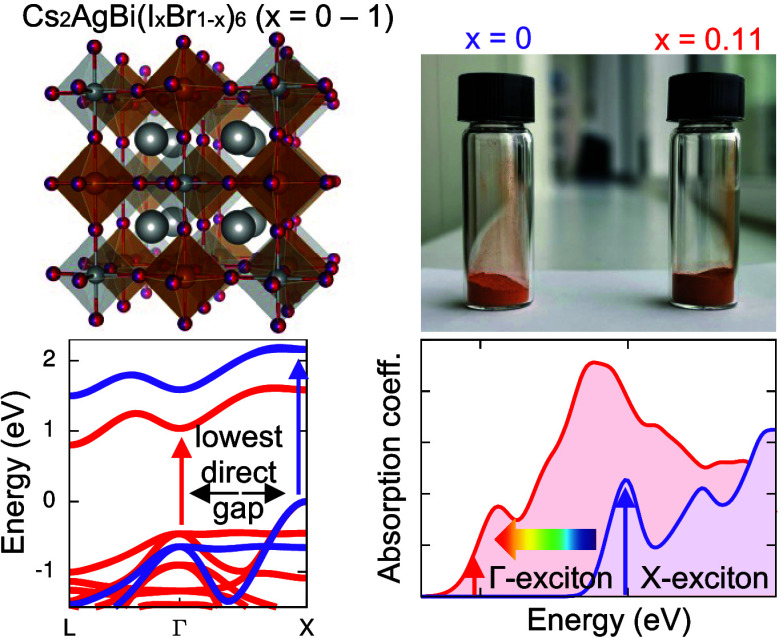

Cs_2_AgBiBr_6_ is an emerging double perovskite semiconductor with robust stability. However, its potential for photovoltaics is limited by its indirect band gap and localized electronic structure featuring a resonant exciton with a large binding energy. Cs_2_AgBi(I_*x*_Br_1–*x*_)_6_ nanocrystals with iodide concentrations of up to 100% were recently demonstrated, but an atomistic understanding of how halide mixing affects the electronic and excited-state structure is missing. Here, we use first-principles GW and Bethe–Salpeter Equation calculations to show that halide mixing leads to a pronounced change in the band gap and character of optical excitations. Exciton binding energies are reduced by up to a factor of 5, with significantly more delocalized excitons in I-rich compounds. We further show that phase-pure bulk alloys with *x* ≤ 0.11 can be fabricated using mechanosynthesis and measure a red-shifted absorption in line with our calculations. Our study highlights that halide mixing in double perovskites can not only lead to significant band gap changes but may also be used for tuning excitonic properties.

## Introduction

Metal-halide perovskites are energy-converting materials for applications ranging from photovoltaics^1,2^ to detectors^3,4^ and photocatalysis.^5^ Halide perovskites with chemical formula ABX_3_, in which A is a (molecular) cation such as methylammonium (MA), formamidinium (FA) or Cs^+^, B is Pb^2+^ or Sn^2+^, and X is a halogen ion, typically I^–^, Br^–^, or Cl^–^, have become particularly relevant as absorber materials in single- and multijunction solar cells.^6−11^

To overcome stability and toxicity limitations of ABX_3_ perovskites and harness a broader range of compositional tunability, halide double perovskites with chemical formula A_2_BB′X_6_ have been widely studied because of their promising optoelectronic properties and stability against thermodynamic degradation and moisture. In particular, the material Cs_2_AgBiBr_6_^12−14^ has a suitable band gap for solar and indoor light absorption^15,16^ and good charge-carrier mobilities.^13^ Despite these favorable properties, photoconversion efficiencies of solar cells based on Cs_2_AgBiBr_6_ have remained ≲6%,^17−20^ which has been linked to the indirect band gap of ∼2.3 eV in combination with limited charge-carrier diffusion lengths,^15,21^ large exciton binding energy,^22−25^ and rapid charge-carrier localization due to strong electron–phonon coupling.^26,27^

At room temperature, Cs_2_AgBiBr_6_ assumes a cubic crystal structure with *Fm*3̅*m* space group.^15^ First-principles electronic structure calculations based on density functional theory (DFT) and the GW approach show that cubic Cs_2_AgBiBr_6_ has a highly anisotropic electronic structure, in which the valence band maximum (VBM)—located at **k**-vector X =  (, 0, ), where *a* is the unit cell length—is primarily derived from Ag d_*z*^2^_, Bi s and Br p orbital contributions, whereas the conduction band minimum (CBM)—at L =  (, , )—stems from Bi p and Ag s contributions.^14,28,29^ This localized and anisotropic electronic structure leads to a resonant, strongly localized exciton with a large binding energy arising from the direct valence band (VB) to conduction band (CB) transition at X and manifests itself as a pronounced particle-like feature in linear optical absorption spectra of this material^30,31^ and other Ag-pnictogen double perovskites.^31,32^ The fine structure and spatial extent of this exciton are ill-described by the hydrogenic Wannier–Mott model,^33^ which assumes isotropic and parabolic band edges and an isotropic and constant dielectric screening on the length scale of the excitonic wave function, a behavior that has also been observed in other double and vacancy-ordered perovskites.^34−37^

Halide mixing is a robust and well-explored strategy for tuning the band gap and other photophysical properties of metal-halide perovskites. In ABX_3_ perovskites, halide mixing has been used to achieve band gaps spanning the entire visible spectrum.^38^ Due to the electronic structure of metal-halide perovskites, which features antibonding halide p states in the valence band maximum, and the size differences between halides with radius *r*_I_ > *r*_Br_ > *r*_Cl_, the band gaps of halide perovskites behave as *E*_I_^gap^ < *E*_Br_^gap^ < *E*_Cl_^gap^.^39^ In ABX_3_ perovskites, the full range of halide mixing ratios can be accessed^38,40^ and band gaps change (nearly) linearly as a function of halide mixing ratio.^40^

In contrast, the synthesis of mixed-halide Cs_2_AgBiX_6_ has proven to be less straightforward. Kubicki et al.^41^ used mechanosynthesis to fabricate mixed-halide Cs_2_AgBiX_6_ with X = I, Br, Cl and showed that mixing Br and Cl retains the cubic double perovskite structure up to 100% Cl but deteriorates the photoluminescence of the material. On the other hand, mixing Br and I, while desirable because of the lower band gap predicted for I-featuring compounds,^42,43^ was reported to produce a range of complex side phases for I ratios exceeding 3 mol %,^41^ including Cs_3_Bi_2_I_9–*x*_Br_*x*_, Cs_3_BiBr_6–*x*_I_*x*_, and other phases that could not be identified in ref (41). However, while bulk Cs_2_AgBiI_6_ remains elusive, this material has recently been reported to form at the nanoscale in refs (44) and (45), in which anion exchange was used to fabricate Cs_2_AgBi(I_*x*_Br_1–*x*_)_6_ nanocrystals, including pure Cs_2_AgBiI_6_ nanocrystals.

Motivated by these reports, we sought to provide an atomistic understanding of how homogeneous halide mixing affects the electronic and excitonic properties of Cs_2_AgBi(I_*x*_Br_1–*x*_)_6_. We use first-principles Green’s function-based many-body perturbation theory in the GW and Bethe–Salpeter Equation (BSE) approaches to calculate band structures and linear optical absorption spectra of cubic Cs_2_AgBi(I_*x*_Br_1–*x*_)_6_ and demonstrate that increasing the I content in this family of materials not only reduces the band gap like in mixed-halide ABX_3_ perovskites but also drastically changes the character of excitons. The pronounced excitonic peak, characteristic of the absorption spectrum of Cs_2_AgBiBr_6_, is suppressed with increasing I content, corresponding to a significant reduction in the exciton binding energy due to an increase in the macroscopic dielectric constant and a reduction in the charge-carrier effective masses. Furthermore, an additional exciton with a binding energy of ∼50 meV, arising primarily from a Γ-point transition, contributes to the onset of absorption for compounds with *x* > 0.5. This exciton is more delocalized and described well by the hydrogenic Wannier–Mott model, in contrast to the exciton arising from electronic transitions at the X point. We complement our first-principles predictions with the experimental characterization of mixed-halide Cs_2_AgBi(I_*x*_Br_1–*x*_)_6_ bulk samples fabricated via mechanochemical synthesis. Optical absorption measurements of samples with *x* ≤ 0.11, which contain no significant amount of side phases, show a red shift of the onset of absorption, in line with our computational predictions. The absorption spectra of samples with *x* > 0.11 are dominated by nonperovskite side phases, highlighting the previously reported importance of nanostructuring and surface stabilization in obtaining iodide-rich Cs_2_AgBi(I_*x*_Br_1–*x*_)_6_ samples. The combination of lower exciton binding energies and reduced and more balanced effective masses could translate into improved generation and transport of charge carriers in I-rich double perovskites. Thus, we demonstrate that halide mixing may be a feasible strategy not only for band gap tuning but also for tailoring the character of excitons in double perovskites.

## Methods

### Computational Methods

We used density functional theory (DFT) as implemented in the plane-wave code quantum espresso(46,47) with the exchange–correlation functional by Perdew, Burke, and Ernzerhof (PBE)^48^ for geometry optimizations and as a starting point for our *G*_0_*W*_0_ calculations. A plane-wave energy cutoff of 60 Ry and a 10 × 10 × 10 Γ-centered **k**-mesh were employed. We used norm-conserving fully relativistic pseudopotentials from the PseudoDojo database,^49,50^ with the following valence electron configurations: Cs 5s^2^ 5p^6^ 6p^1^, Ag 4s^2^ 4p^6^ 4d^10^ 5s^1^, Bi 5d^10^ 6s^2^ 6p^3^, Br 4s^2^ 4p^5^, and I 5s^2^ 5p^5^. Spin–orbit coupling (SOC) was taken into account self-consistently for all calculations unless otherwise noted.

We modeled the structure of cubic Cs_2_AgBiBr_6_ starting from the experimental room-temperature crystal structure reported in ref (13). The alloyed systems Cs_2_AgBi(I_*x*_Br_1–*x*_)*_6_* were represented in the primitive unit cell with *Fm*3̅*m* space group using the virtual crystal approximation (VCA)^51^ and assuming homogeneous mixing of Br and I. The atomic positions and lattice parameters of all structures were fully relaxed using DFT–PBE. The convergence criteria for the geometry optimization are 10^–8^ Ry for the total energy and 10^–6^ Ry/Bohr for forces on atoms. A plane-wave energy cutoff of 60 Ry and a 10 × 10 × 10 Γ-centered **k**-mesh was used. All relaxations were performed without accounting for SOC.

For the calculation of quasiparticle band gaps and band structures, we used the relaxed crystal structures and the one-shot *G*_0_*W*_0_ approach, in which we constructed the zeroth-order Green’s function *G*_0_ and screened Coulomb interaction *W*_0_ from DFT eigenvalues and eigenfunctions calculated using the PBE exchange–correlation functional. For the *G*_0_*W*_0_ + BSE calculations, we used the BerkeleyGW code.^52,53^ The dielectric screening and quasiparticle band gap of all materials was computed using a polarizability cutoff of 8 Ry, the generalized plasmon-pole model by Godby and Needs,^54^ an energy cutoff of 48 Ry for the bare Coulomb interaction, and 600 bands. With these settings, we report band gaps converged to within 0.1 eV (see Supporting Information for convergence tests; Figure S7 for Cs_2_AgBiBr_6_ and Figure S8 for Cs_2_AgBiI_6_, respectively). *G*_0_*W*_0_@PBE band structures were obtained through Wannier interpolation using the wannier90 code.^55^ Effective masses were calculated as second derivatives of the energy bands using finite differences as described in the Supporting Information of ref (31).

Optical spectra and excitonic properties were computed by solving the Bethe–Salpeter equation (BSE)^56−61^ with the Tamm–Dancoff approximation (TDA).^61^ We obtain linear optical absorption spectra from the imaginary part of the transverse dielectric function using the momentum operator formulation. We constructed the electron–hole interaction kernel *K*^eh^ on a 4 × 4 × 4 **k**-point grid, using a set of 22 valence and 22 conduction bands. All absorption spectra were obtained by interpolating the electron–hole kernel on a fine 14 × 14 × 14 **k**-point grid and applying a constant arbitrary Gaussian smearing of 50 meV. For the fine grid interpolation, we used 4 valence and 6 conduction bands. With these settings, we report exciton binding energies converged to within 5 meV (see convergence tests in Figure S11 of the Supporting Information). Here, and unless otherwise noted, the exciton binding energies are defined as the absolute difference between the computed excitation energy and the lowest-energy direct transition from which the excited state originates.

All computational settings are summarized in Table S1.

### Chemicals and Mechanosynthesis

99% cesium bromide (CsBr, Tokyo Chemical Industries), 99.0% cesium iodide (CsI, Tokyo Chemical Industries), 99.998% silver bromide (AgBr, Alfa Aeser), 99.999% silver iodide (AgI, Permion), 99% bismuth bromide (BiBr_3_, Alfa Aeser), and 99% bismuth iodide (BrI_3_, Sigma-Aldrich). Prior to the synthesis, BiBr_3_ was dried at 70 °C under vacuum.

A stochiometric mixture of CsBr, AgBr, BiBr_3_, CsI, AgI, and BiI_3_ (ca. 2 g in total) was loaded in a 10 mL stainless steel ball mill jar filled with two stainless steel beads (10 mm in diameter). The double perovskite compositions were synthesized by milling at 30 Hz for 90 min using a Retsch MM500 vario ball mill. All compositions were annealed at 150 °C for 30 min.

### X-ray Diffraction

X-ray diffraction experiments were performed using a Bruker D8 Advance equipped with a Cu Kα_1,2_ (λ = 1.54184 Å) radiation source operating at 40 kV and 40 mA. The patterns were recorded by measuring in Bragg–Brentano geometry at diffraction angles from 5 to 60° 2θ, with a step size of 0.02° and an integration time of 0.5 s.

Rietveld refinements were performed using the FullProf Suite software with the crystallographic information files of AgI (ICSD entry 56552), Cs_3_Bi_2_Br_9_ (ICSD entry 1142), and Cs_2_AgBiBr_6_ (ICSD entry 252164) as input files. For the fitting procedure, a pseudo-Voigt function was fit to the experimentally obtained reflections. Moreover, a scaling factor, the lattice parameters, shape factor, and a factor accounting for the reflection width were used as fitting variables.

### Diffuse Reflectance Spectra

The absorption profile was approximated by recording the diffuse reflectance spectrum and the Kubelka–Munk relation. The diffuse reflectance was recorded by using a PerkinElmer LAMBDA 950S UV/vis/NIR spectrometer equipped with an integrating sphere. The reflectance was recorded with respect to a poly(tetrafluoroethylene) (PTFE) background from 900 to 350 nm (2 nm step size and 0.4 s integration time).

## Results and Discussion

**Figure 1:**
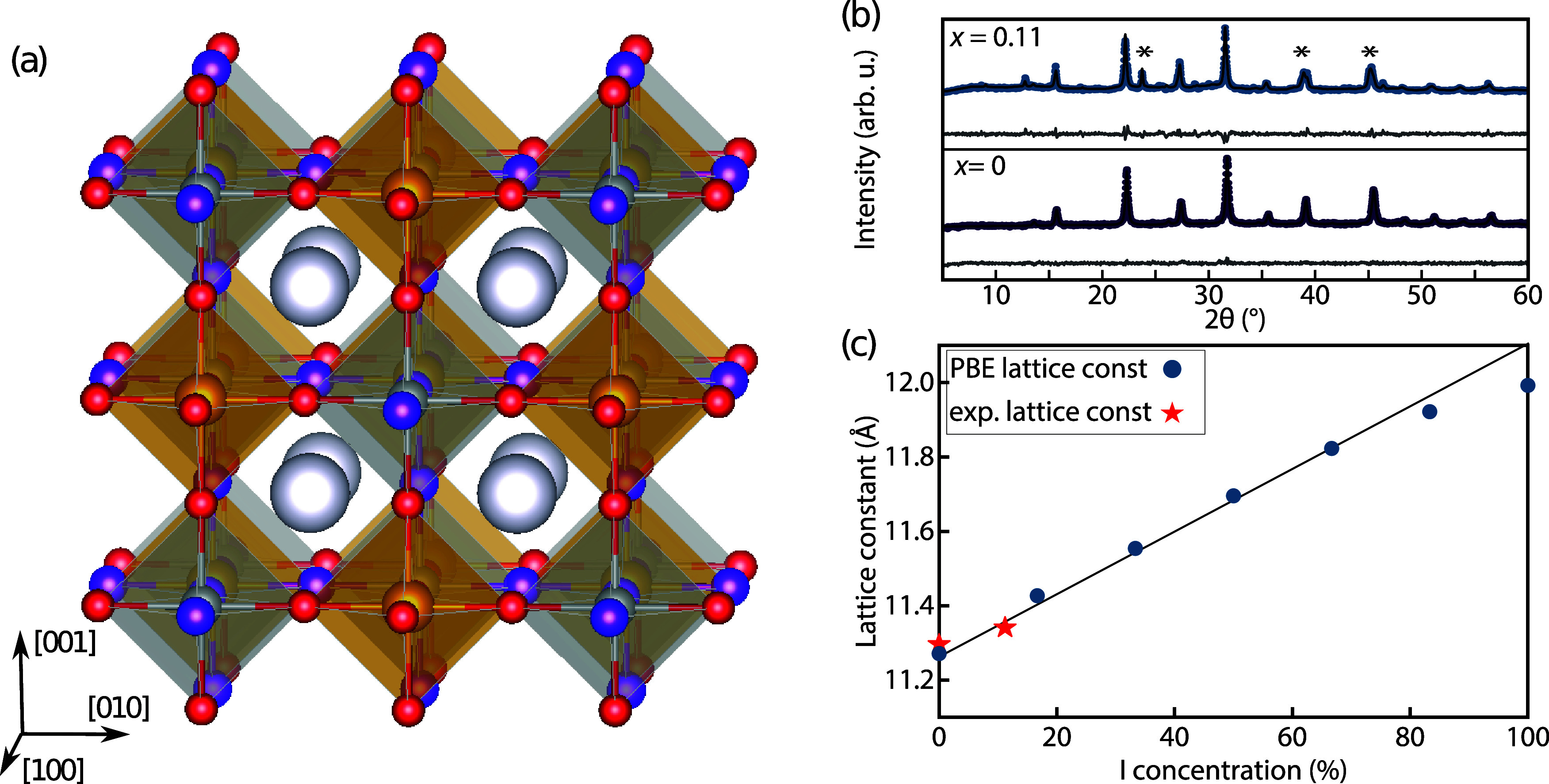
(a) Three-dimensional (3D) representation of the cubic crystal structure of Cs_2_AgBi(I_*x*_Br_1–*x*_)_6_, where silver balls represent Ag, orange Bi, white Cs, and red and purple I and Br, respectively. (b) Experimental X-ray diffraction patterns of Cs_2_AgBi(I_*x*_Br_1–*x*_)_6_ with *x* = 0 and 0.11 and the corresponding Rietveld refinement fit (black) and residual (gray). For *x* = 0.11, unreacted AgI is still observed in the diffraction pattern and indicated with an asterisk. (c) DFT–PBE (blue, dots) and experimentally (red, star) determined lattice constant, *a*, as a function of iodide concentration. The black line corresponds to the trendline for Cs_2_AgBi(I_*x*_Br_1–*x*_)_6_ nanocrystals and is reproduced from ref (45). Copyright 2023 American Chemical Society.

We start by investigating the structural changes in the crystal lattice of cubic Cs_2_AgBiBr_6_ (Figure 1(a)) upon incorporation of I. To this end, we perform structural optimizations with DFT using the exchange–correlation functional of Perdew, Burke, and Ernzerhof (PBE)^62^ as implemented in quantum espresso.^63^ We represent the mixed halide sites using the virtual crystal approximation,^51^ i.e., by replacing the X (halide) site with a “virtual atom”, which constitutes an interpolation between the pure halide sites X = Br and X = I. This approximation relies on the assumption of a homogeneous distribution of Br and I in the material that does not lead to pronounced local structural distortions and nonlinear mixing effects such as band gap bowing. It has been used for modeling the structural and optoelectronic properties of halide perovskites with DFT^64,65^ and in conjunction with the GW + BSE method for understanding optical properties of mixed CsPb(Br_*x*_I_1–*x*_)_3_ in excellent agreement with experimental results.^40^ We note that these calculations by definition do not account for local structural distortions, phase segregation, or the formation of side phases. Rather, we deliberately model homogeneously mixed alloys of Cs_2_AgBi(I_*x*_Br_1–*x*_)_6_ and provide a discussion of the validity of the VCA in the SI (see Figures S1–S5). The DFT–PBE lattice parameters (Table 1) exhibit a nearly linear dependence on I concentration, as shown in Figures 1(c) and S2, in agreement with Vegard’s law^66^ and the trends reported in ref (45) for Cs_2_AgBi(I_*x*_Br_1–*x*_)_6_ nanocrystals. On experimentally replacing Br with I, we observe a shift of the X-ray diffraction peaks to lower angles (Figure 1(b)), in line with lattice expansion due to incorporation of the larger I (see SI for experimental procedures). From Rietveld refinement of the experimentally obtained X-ray diffraction patterns, we observe successful halide mixing up to *x* ≤ 0.11 in the mechanochemically synthesized double perovskites. We estimated *x* using the refined lattice parameters and the sizing curve reported for mixed-halide Cs_2_AgBi(I_*x*_Br_1–*x*_)_6_ nanocyrstals.^45^ Note that this I/Br ratio is higher than previously reported for bulk compounds,^41^ but lower than feasible in nanocrystals. The higher degrees of iodide substitution in colloidal nanocrystals, compared with bulk compounds, have been attributed to thermodynamic stabilization. This stabilization is achieved through the increased importance of surface free energy at the nanoscale and the role of stabilizing surface ligands, as discussed in ref (45). Next to the double perovskite phase, Rietveld refinement fits reveal a minor presence of the typically observed silver-free phase Cs_3_Bi_2_(Br_1–*x*_I_*x*_)_9_ for *x* = 0.11, which shows a characteristic reflection at 12.76°and becomes the dominating phase for larger iodide concentrations as shown in Figures 1(b) and S6.

**Table 1:** Computed Lattice Parameters (in Å), Static Dielectric Constant (ε_∞_), QP Band Gap (in eV) Indirect from X^VBM^ → L^CBM^, Lowest-Energy Direct Transition (in eV), and the High-Symmetry **k**-Point at Which It Is Found of Mixed Halide Double Perovskites Cs_2_AgBi(I_*x*_Br_1–*x*_)_6_

			QP band gap (eV)	lowest direct transition	
mixing ratio	lattice constant (Å)	dielectric constant (ε_∞_)	X^VBM^ → L^CBM^	energy (eV)	*k*-point
0.00	11.27	5.41	1.50	2.16	X
0.16	11.43	5.77	1.34	2.03	X
0.33	11.55	6.16	1.19	1.91	Γ ≡ X
0.50	11.70	6.49	1.08	1.81	Γ ≡ X
0.66	11.82	6.83	0.98	1.70	Γ
0.83	11.92	7.20	0.89	1.60	Γ
1.00	11.99	7.61	0.80	1.50	Γ

**Figure 2:**
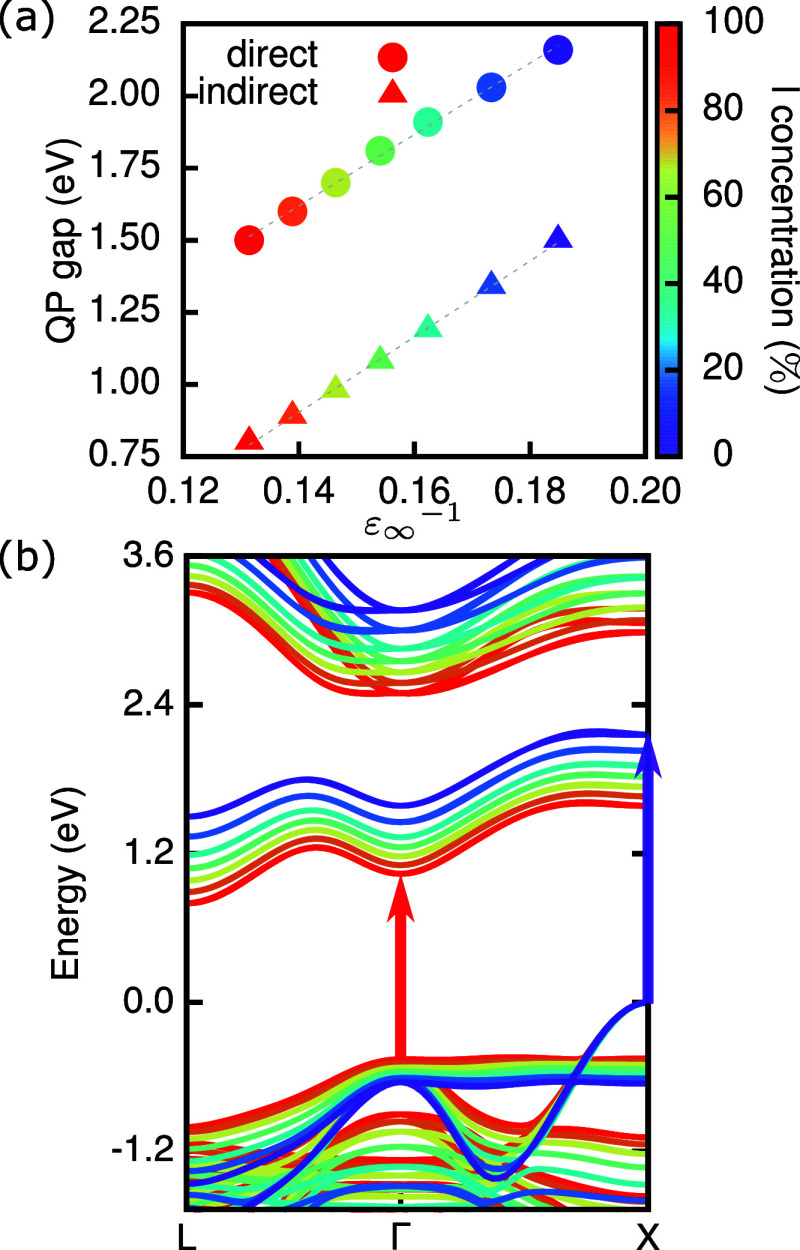
Electronic properties of mixed halide double perovskites Cs_2_AgBi(I_*x*_Br_1–*x*_)_6_. (a) QP band gap (indirect, triangle) and lowest-energy direct transition (circles); (b) QP band structure aligned to the top of the valence band, which is chosen as the zero of the energy scale.

Next, we compute the band structure and quasiparticle (QP) band gap of the double perovskite series Cs_2_AgBi(I_*x*_Br_1–*x*_)_6_ within the *G*_0_*W*_0_ approach,^56^ in which QP eigenvalues are calculated by perturbatively correcting the DFT–PBE Kohn–Sham eigenvalues. We take spin–orbit coupling into account self-consistently.^67^Figure 2(a) shows the fundamental (indirect) and lowest-energy direct gap. Note that our calculations consistently underestimate experimental band gaps as reported before and attributed to the dependence of the *G*_0_*W*_0_ band gap on the DFT “starting point”.^31,68,69^ Both band gaps decrease linearly with increasing I content as expected and reported previously.^43^ We further find a linear relationship between these band gaps and the static dielectric constant, which increases with an increasing I content (see Figure S9 and Table 1).

In Figure 2(b), we show the change in the QP band structure upon increasing the I concentration (i.e., from purple to red). I-rich compounds feature a smaller band gap and a reduction in the bandwidth, with a more pronounced effect in the valence manifold. Importantly, with an increase in *x*, we observe a change in the **k**-vector of the lowest direct band gap. In Cs_2_AgBiBr_6_, the lowest transition is at the X point of the Brillouin zone. At this point, the valence band has Bi s and Ag d_*z*^2^_ character, while the conduction band is derived from Bi p contributions (see Figure S10(a)). Both valence and conduction bands are highly anisotropic at the X point and we have shown previously, that this leads to a localized resonant exciton in Cs_2_AgBiBr_6_ and related materials.^31^ In compounds with *x* > 0.5, including Cs_2_AgBiI_6_, the lowest direct transition is shifted to the Γ point, where the valence band is almost entirely derived from I p orbital contributions, while the conduction band still has contributions predominantly from Bi p, but it is much more disperse (see Figure S10(b)).

In order to quantify this change in band edge anisotropy, we computed effective masses at the **k**-vector of the lowest-energy direct transition. In Table 2, we report the *G*_0_*W*_0_ effective masses and the corresponding anisotropy factors , where m and m are transverse and longitudinal effective masses, respectively. The electron effective masses of the I-rich compounds, i.e., with mixing ratios *x* > 0.5, are at least 3.5 times smaller than that of the pure Cs_2_AgBiBr_6_ perovskite. This is a result of the shift in the position of the lowest-energy direct transition to Γ, where the conduction band is more disperse. Another consequence of the shift is the presence of light and heavy holes in the electronic structures of the mixed halide perovskites with *x* > 0.5. The hole effective mass is the average between the two and, therefore, the I-rich compounds also feature smaller hole effective masses than their Br counterparts. Consequently, the reduced effective mass of the I-rich double perovskites is almost half of that of the pure Br material.

**Table 2:** *G*_0_*W*_0_ Hole (*m*_h_^*^), Electron (*m*_e_^*^), and Reduced (μ) Effective Masses (in Units of Electron Rest Mass *m*_0_) and the Corresponding Anisotropy Factors (λ_h_, λ_e_, and λ_μ_, Respectively) for the Studied Br/I Mixing Ratios

mixing ratio	effective masses			anisotropy factor		
*x*	*m*_h_^*^	*m*_e_^*^	μ	λ_h_	λ_e_	λ_μ_
0.00	0.202	0.579	0.150	0.54	0.57	0.55
0.16	0.192	0.489	0.138	0.53	0.54	0.56
0.33	0.179	0.418	0.126	0.53	0.55	0.54
0.50	0.181	0.390	0.124	0.53	0.56	0.54
0.66	0.135	0.162	0.074	0.64	0.65	0.64
0.83	0.141	0.159	0.075	0.64	0.65	0.65
1.00	0.144	0.155	0.074	0.64	0.64	0.64

**Figure 3:**
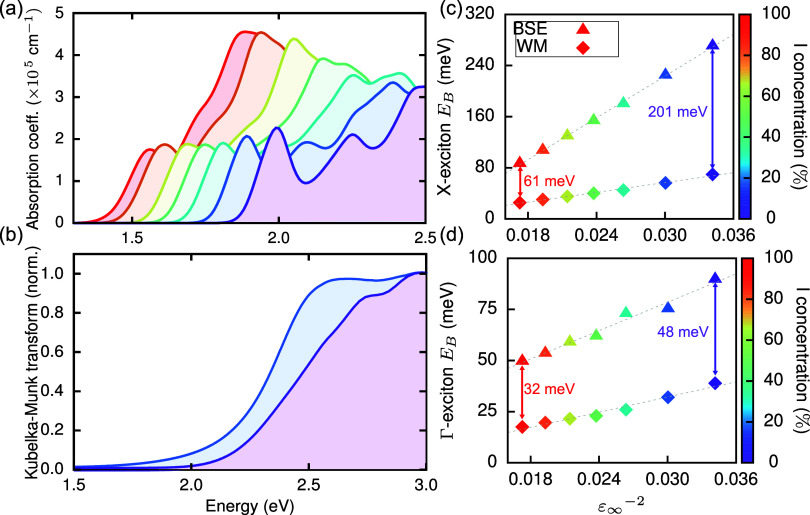
Optical properties of mixed halide double perovskites Cs_2_AgBi(I_*x*_Br_1–*x*_)_6_. The color scale denotes the I concentration, i.e., *x* ·100. (a) Linear optical absorption spectra of Cs_2_AgBi(I_*x*_Br_1–*x*_)_6_ as computed within *G*_0_*W*_0_@PBE + BSE. (b) Kubelka–Munk transform of the diffuse reflectance spectra of Cs_2_AgBi(I_*x*_Br_1–*x*_)_6_ samples with *x* = 0 and 0.11. (c, d) Computed exciton binding energies as predicted by the Wannier–Mott model (squares) and BSE binding energy of the exciton arising from direct transitions at (c) X and (d) Γ, respectively.

In Figure 3(a), we show that the observed changes in the electronic band structure lead to pronounced variations in the linear optical absorption spectra of Cs_2_AgBi(I_*x*_Br_1–*x*_)_6_ as *x* is increased. We calculate these absorption spectra using the BSE approach,^70^ which includes electron–hole interactions and allows for insights into excitonic effects for a wide range of materials.^57,−77^ Note that we here consider only direct excitations with zero momentum transfer. In line with the decrease of the QP band gap, we observe a red shift in the absorption spectra with increasing I content. Furthermore, the excitonic peak originating from the direct transition at X^24,30,31^ is increasingly less pronounced, and the binding energy of the exciton constituting this peak decreases with increasing I concentration (Figure S12). In compounds with *x* > 0.5, in which the lowest direct transition arises from Γ, we find five dark and three degenerate bright excitations with relatively low oscillator strength below this excitonic peak, which arise from the lowest-energy direct transition at Γ (Table S3 and Figure S13). Our calculated absorption spectra qualitatively agree with those reported for Cs_2_AgBi(I_*x*_Br_1–*x*_)_6_ nanocrystals. These nanocrystals exhibit a broadening of the absorption onset and a suppression of the excitonic peak with increasing iodide substitution.^44^

In line with our predictions, we also observe a red shift in the experimentally determined absorption profiles of Cs_2_AgBi(I_*x*_Br_1–*x*_)_6_ bulk powders with *x* ≤ 0.11 (Figure 3(b)), indicating a reduction of the band gap energy. Our experimental absorption profiles were obtained from powder samples using diffuse reflectance spectroscopy and approximated with the Kubelka–Munk transform. As a result, they do not show the pronounced excitonic feature seen in previous experiments on thin films.^24^ Attempts to synthesize thin-film samples with *x* = 0.11 resulted in low-quality films that were unsuitable for absorption experiments. Therefore, our experimental results cannot confirm excitonic effects but do verify the red shift of the absorption onset predicted by our calculations and observed in nanocrystal experiments.^44^ The shallow slope at the absorption onset suggests the indirect band gap is maintained on exchanging less than ∼11% of Br with I. For higher I concentrations (*x* > 0.11), a steeper slope in the absorption profiles is observed, suggesting a change to a more direct band gap transition (Figure S14b). However, control experiments on the silver-free phase, i.e., Cs_3_Bi_2_(Br_1–*x*_I_*x*_)_9_, confirm that the absorption profile for *x* = 0.33 is dominated by the silver-free phase (Figure S14c), whose absorption onset is dominated by a pronounced excitonic feature too, as confirmed by previous first-principles calculations.^78^

In our previous work, we showed that the non-hydrogenic character of excitons in Cs_2_AgBiBr_6_ and other double perovskites is intimately linked to the anisotropic electronic structure and dielectric screening in these materials,^31,36^ as a consequence of the relatively large and anisotropic effective masses at X.^29^ Here, we show that increasing the I concentration has two distinct effects on excitons in these compounds. For this purpose, we define X- and Γ-excitons, as the lowest-energy excitons arising from transitions at X and Γ, respectively. Their binding energies, *E*_B_, are shown in Figure 3(c,d), respectively. First, we find that the binding energy of the X- and Γ-excitons, calculated with respect to the direct QP transition at X and Γ, respectively, decreases with increasing I concentration. The decrease is linear in 1/ε_∞_^2^ as expected, albeit with different slopes. In particular, the binding energy of the X-exciton decreases drastically, by a factor of 5, from *x* = 0 to 1. Second, the character of both excitons also changes, which we demonstrate by comparing our first-principles results with exciton binding energies computed using the Wannier–Mott model,^33^ i.e., by assuming a hydrogenic series of exciton states with binding energies , where *R*_H_ is the Rydberg constant, *n* is the principal quantum number, and values for the reduced effective mass μ and dielectric constant ϵ_∞_ are obtained from our *G*_0_*W*_0_ calculations (Tables 1 and 2). The exciton binding energies of the 1s excitons calculated using the Wannier–Mott model are also shown in Figure 3(c,d). We find that the X-exciton of Cs_2_AgBiBr_6_ has a non-hydrogenic fine structure and a binding energy ∼200 meV larger than predicted by the Wannier–Mott model. As the I concentration increases, the deviation from the Wannier–Mott model becomes significantly smaller. Additionally, we observe that the Γ-exciton is in much better agreement with the Wannier–Mott model across all I concentrations with an absolute deviation of only ∼30 meV for Cs_2_AgBiI_6_. This is in line with the differences in the orbital character at the VBM and CBM at X and Γ, which leads to significantly more disperse and isotropic band edges at Γ (Table S2).

**Figure 4:**
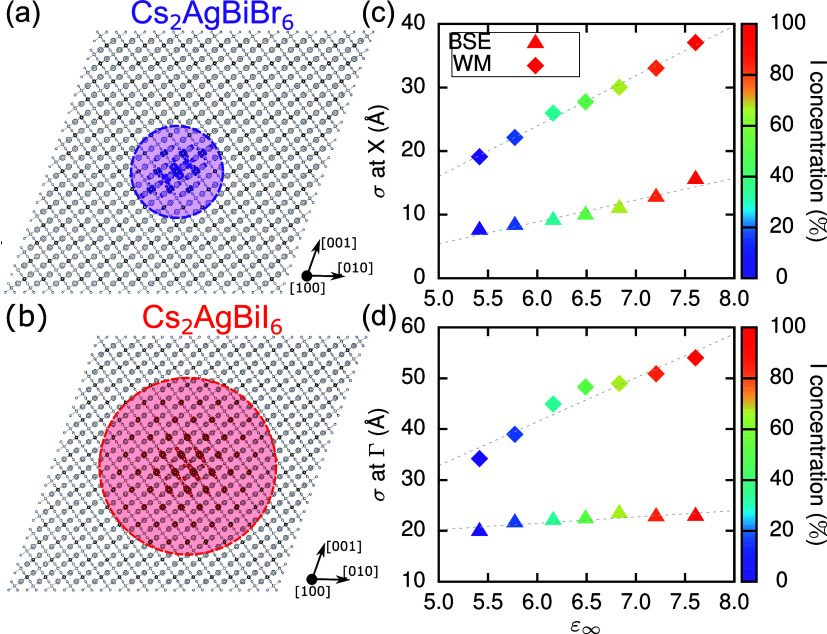
3D representation of the probability density of the exciton wave function in real space, depicted as purple isosurfaces for (a) Cs_2_AgBiBr_6_ and red isosurfaces for (b) Cs_2_AgBiI_6_, showing 95% of the maximum isovalue. (c, d) Exciton localization as defined in the SI and calculated using *G*_0_*W*_0_@PBE + BSE (triangles) and using the Wannier–Mott model calculated for the excitons at (c) X and (d) Γ. The gray dashed lines are linear fits denoting the linear relationship between exciton radius and dielectric constant expected from the Wannier–Mott model.

This pronounced change in exciton character is also reflected in the spatial extent of the X- and the Γ-exciton. We investigate this effect by evaluating the probability distribution of the excitonic wave function Ψ_S_(**r**_e_,**r**_h_) = ∑_*vc***k**_*A*_*vc***k**_^S^ψ_*c***k**_(**r**_e_) ψ_vk_^*^(**r**_h_), where ψ_*v*(*c*) **k**_(**r**_h(e)_) are single-particle DFT–PBE wave functions of electrons and holes, and *A*_*vc***k**_^S^ are coefficients corresponding to the excitonic state S, obtained by solving the BSE. We systematically sample three different hole positions—on Ag, on Bi, and the halide—sum the corresponding probability distributions and plot the resulting excitonic wave function of the pure compounds in Figure 4(a,b) for Cs_2_AgBiBr_6_ and Cs_2_AgBiI_6_, respectively. This comparison shows that the X-exciton of Cs_2_AgBiBr_6_ is significantly more localized than the Γ-exciton of Cs_2_AgBiI_6_, which is in line with its less hydrogenic character.

Furthermore, we quantify the spatial extent of the exciton by calculating the average electron–hole separation, σ, using the approach introduced in ref (74) and as used before in ref (31) (see SI for details). In Figure 4(c,d), we show σ and compare it with the Wannier–Mott radius for the X- and Γ-exciton, respectively (see also Table S3). We find that the average electron–hole separation of the X-exciton is significantly smaller than the corresponding Wannier–Mott radii, which is in line with our earlier observations. Furthermore, it increases by a factor of 2 upon increasing the I content from *x* = 0 to 1. The Γ-exciton shows a much smaller variation as a function of I concentration and reaches a maximum average electron–hole separation of ∼20 Å in Cs_2_AgBiI_6_.

## Conclusions

In conclusion, we predict a significant change in the optoelectronic properties of Cs_2_AgBiBr_6_ upon introduction of I, in particular a reduction of the band gap and the exciton binding energy and a pronounced change of the character of the exciton which becomes more delocalized and “hydrogenic”. We correlate the change in exciton character to a change in the **k**-vector at which the lowest direct transition, responsible for the onset of absorption, takes place. For *x* > 0.5, this transition is at the Γ point, at which valence and conduction band edges are more isotropic and disperse than at the X point, from which the lowest direct transition arises in Br-rich compounds. Experimentally, our prediction of a band gap red shift is confirmed in bulk Cs_2_AgBi(I_*x*_Br_1–*x*_)_6_ with *x* up to 0.11. Our findings show that halide mixing can be a powerful method for tuning not only band gaps of halide perovskites but also their excitonic properties in chemically heterogeneous double perovskites. The reduced band gap, lower exciton binding energies, less confined excitons, and lower effective masses are favorable for photovoltaic performance, and motivate further efforts to experimentally obtain phase-pure Cs_2_AgBiI_6_ in bulk form.

## Supporting Information Available

Additional data on structural and optical characterization, computational methods, information about convergence tests and data supporting the validity of the virtual crystal approximation (PDF)
