# Supplementary material for: Halide Mixing in Cs2AgBi(IxBr1–x)6 Double Perovskites: A Pathway to Tunable Excitonic Properties
Source: J Phys Chem C Nanomater Interfaces. 2024 Aug 26;128(35):14767–75. doi: 10.1021/acs.jpcc.4c04453 (PMC11382272; doi:10.1021/acs.jpcc.4c04453)
Supplement: Supplementary file 1 — jp4c04453_si_001.pdf [file jp4c04453_si_001.pdf]

Supporting Information for Halide Mixing in $Cs_2AgBi(I_xBr_{1-x})_6$ Double Perovskites: A Pathway to Tunable Excitonic Properties

Raisa-Ioana Biega,† Huygen J. Jöbsis,‡ Zamorano Gijsberg,‡ Maxim Hüskens,†
Eline M. Hutter,‡ and Linn Leppert*,†

†*MESA+ Institute for Nanotechnology, University of Twente, 7500 AE Enschede, The Netherlands*

‡*Debye Institute for Nanomaterials Science, Department of Chemistry, Princetonlaan 8, 3584 CB Utrecht, the Netherlands*

E-mail: l.leppert@utwente.nl

Validity of the Virtual Crystal Approximation

We carefully tested the validity of the Virtual Crystal Approximation (VCA) by comparing lattice parameters, bond lengths, bandstructures and band gaps to calculations in which halide mixing was explicitly modeled. For these calculations, we used the VASP program package[1] with projector augmented wave (PAW) potentials[2] with the following valence electron configurations: Cs $5s^2$ $5p^6$ $5d^1$, Ag $4d^{10}$ $5s^1$, Bi $6s^2$ $6p^3$, Br $4s^2$ $4p^5$, I $5s^2$ $5p^5$. We used a cutoff energy of 600 eV for the plane-wave basis set and a **k**-grid of $6 \times 6 \times 6$ to achieve an equivalent level of convergence in these calculations as with QUANTUM ESPRESSO. In all geometry optimizations, forces were converged to 0.01 eV/Å. All calculations were carried out using the primitive unit cell. We note that the valence electron configuration of the PAW potentials used in our VASP calculations differs from the one of the norm-conserving pseudopotentials we used in QUANTUM ESPRESSO. In the latter we used pseudopotentials including semicore electrons for Ag and Bi. This leads to two main differences in the bandstructure of $Cs_2AgBiBr_6$ as calculated with DFT-PBE, as shown in Figure S1. First, the band gap is ∼0.4 eV smaller with QUANTUM ESPRESSO as compared to VASP which is in line with previous studies highlighting the importance of including semicore states for band gaps of halide perovskites.[3–5] Second, the highest-energy valence band has a slightly larger dispersion in our QUANTUM ESPRESSO calculations which leads to a larger energy difference between the direct band gaps at Γ and L than in VASP, and nearly degenerate direct band gaps at Γ and L for I-rich compounds predicted in VASP.

Lattice parameters are shown in Figure S2 and demonstrate that explicit modeling of these mixed compositions leads to a linear increase of lattice parameters with increasing I content in excellent agreement with the VCA results. Since we used the primitive unit cell for all calculations, explicit structural models feature large distortions and lattice vectors of different lengths, deviating from the on-average cubic structure of our VCA models and at odds with our experimental results for $x = 0.11$. Nonetheless, average lattice vectors are in excellent agreement with each other. Furthermore, we observe similar trends for Bi-halide

and Ag-halide bond lengths, which we show in Figure S3.

The structural distortions present in the explicit structural models, lead to small differences in the bandstructures as compared with those calculated using the VCA, as shown in Figure S4 and consequently in the band gaps shown in Figure S5, which are more pronounced for the lowest-energy direct band gaps (Figure S5b) than for the indirect band gaps (Figure S5a) due to subtle differences in band dispersion resulting from the pronounced structural distortions in the explicit structural models. These differences in bandstructures and band gaps are expected to be smaller in explicit structural models using larger unit cells. Overall, while the VCA has limitations for modelling local structural distortions, phase segregation and the formation of side phases due to halide mixing, our results suggest that it captures the electronic structure of homogeneously mixed $Cs_2AgBiI_xBr_{6-x}$ double perovskites.

Calculation of exciton localization

In order to quantify the spatial extent of the exciton, we employ an approach inspired by Ref. 6, and used before in Ref. 7, and define the electron–hole correlation function $F_S(\mathbf{r}) = \int_\Omega d^3\mathbf{r}_h |\Psi_S(\mathbf{r}_e = \mathbf{r}_h + \mathbf{r}, \mathbf{r}_h)|^2$. $F_S(\mathbf{r})$ provides the probability of finding electron and hole pair separated by the vector $\mathbf{r} = \mathbf{r_e} - \mathbf{r_h}$. We compute the integral as a discrete sum over $\mathbf{r}_h$, with $\mathbf{r}_h$=$\mathbf{r}(\mathrm{Ag}^+)$, $\mathbf{r}(\mathrm{Bi}^{3+})$ and $\mathbf{r}(\mathrm{X}^-)$. To approximately account for the symmetry and finite number of hole positions, we introduce the weight w_h and normalize $F_S(\mathbf{r})$ with respect to its cumulative sum:

$$F_S(\mathbf{r}) = \frac{\sum_h \left(|\Psi_S(\mathbf{r}_e = \mathbf{r}_h + \mathbf{r}, \mathbf{r}_h)|^2 \cdot w_h\right)}{\sum_{e,h} \left(|\Psi_S(\mathbf{r}_e, \mathbf{r}_h)|^2 \cdot w_h\right)} \tag{S1}$$

where $w_h = \begin{cases} 6 & \text{for } \mathbf{r}_e = \mathbf{r}(\mathrm{X}^-) \\ 1 & \text{otherwise} \end{cases}$. Using the distribution function defined in expression S1, we compute the average electron-hole separation $\sigma_{\mathrm{BSE}} = \sqrt{\langle |\mathbf{r}|^2 \rangle - \langle |\mathbf{r}| \rangle^2}$, where $\langle |\mathbf{r}|^n \rangle =$

$\int_\Omega d^3\mathbf{r}|\mathbf{r}|^n F_S(\mathbf{r})$, and use it to quantify the degree of localization of the excitonic wave function (see Table S2).

Table S1: Computational settings for calculations of optoelectronic properties.

Theory level	Input parameter	Value
DFT	cutoff energy	60 Ry
	**k**-point grid	$10 \times 10 \times 10$
GW	cutoff energy for ε	8 Ry
	cutoff energy for Σ	48 Ry
	total number of bands	600
BSE	states coarse grid	22 occupied<br>22 unoccupied
	states fine grid	4 occupied<br>6 unoccupied
	coarse grid	$4 \times 4 \times 4$
	fine grid	$14 \times 14 \times 14$
	smearing	50 meV

Table S2: Exciton binding energy (in meV) of first dark and first bright transitions as computed with G_0W_0@PBE+BSE $E_{\mathbf{BSE}}$, the Wannier–Mott model[8] $E_{\mathbf{WM}}$, and the Wannier–Mott model including effective mass anisotropy[9] $E_{\mathbf{WM}}(\lambda)$.

Mixing ratio	BSE exciton binding energy (meV)				Wannier-Mott model	
x	$E_{\mathrm{BSE}}^{\mathrm{dark}}$	**k**-point	$E_{\mathrm{BSE}}^{\mathrm{bright}}$	**k**-point	E_{WM}	$E_{\mathrm{WM}}(\lambda)$
0.00	260.40	X	181.25	X	69.58	48.87
0.16	224.87	X	154.83	X	56.35	39.78
0.33	180.23	X	122.39	X	44.99	31.53
0.50	153.83	X	104.90	X	39.99	28.07
0.66	129.81	Γ	57.59	Γ	21.51	15.65
0.83	53.55	Γ	53.26	Γ	19.64	14.33
1.00	49.69	Γ	49.36	Γ	17.52	12.73

Table S3: G_0W_0**@PBE+BSE exciton localization** ($\sigma_{\mathbf{BSE}} = \sqrt{< \mathbf{r}^2 > - < \mathbf{r} >^2}$**, in Å) and binding energy (in meV) and of the exciton arising from direct transitions at** Γ ($E^{\Gamma}_{\mathbf{BSE}}$) **and at X** ($E^{\mathbf{X}}_{\mathbf{BSE}}$)**. Wannier–Mott model predictions for exciton binding energy** ($E_{\mathbf{WM}} = \frac{\mu}{m_0 \varepsilon_\infty^2} R_H$) **and exciton extent in real space** ($r_{\mathbf{WM}} = \frac{m_0 \varepsilon_\infty}{\mu} a_H$)**.**

**Mixing ratio** x	**k-point**	**Exciton binding energy (meV)** E_{BSE}	E_{WM}	**Exciton localization (Å)** σ_{BSE}	r_{WM}
0.00	X	270.71	69.58	7.52	19.12
	Γ	86.76	38.89	19.85	34.20
0.16	X	224.87	56.35	8.28	22.14
	Γ	82.05	32.01	21.55	38.97
0.33	X	180.23	44.99	9.08	25.97
	Γ	79.98	26.00	22.00	44.94
0.50	X	153.83	39.99	9.91	27.74
	Γ	61.95	22.97	22.33	48.29
0.66	X	129.81	35.07	10.96	30.05
	Γ	59.02	21.51	23.39	48.99
0.83	X	107.28	30.24	12.71	33.05
	Γ	53.55	19.64	22.73	50.87
1.00	X	86.78	25.53	15.51	37.08
	Γ	49.69	17.52	22.82	54.03

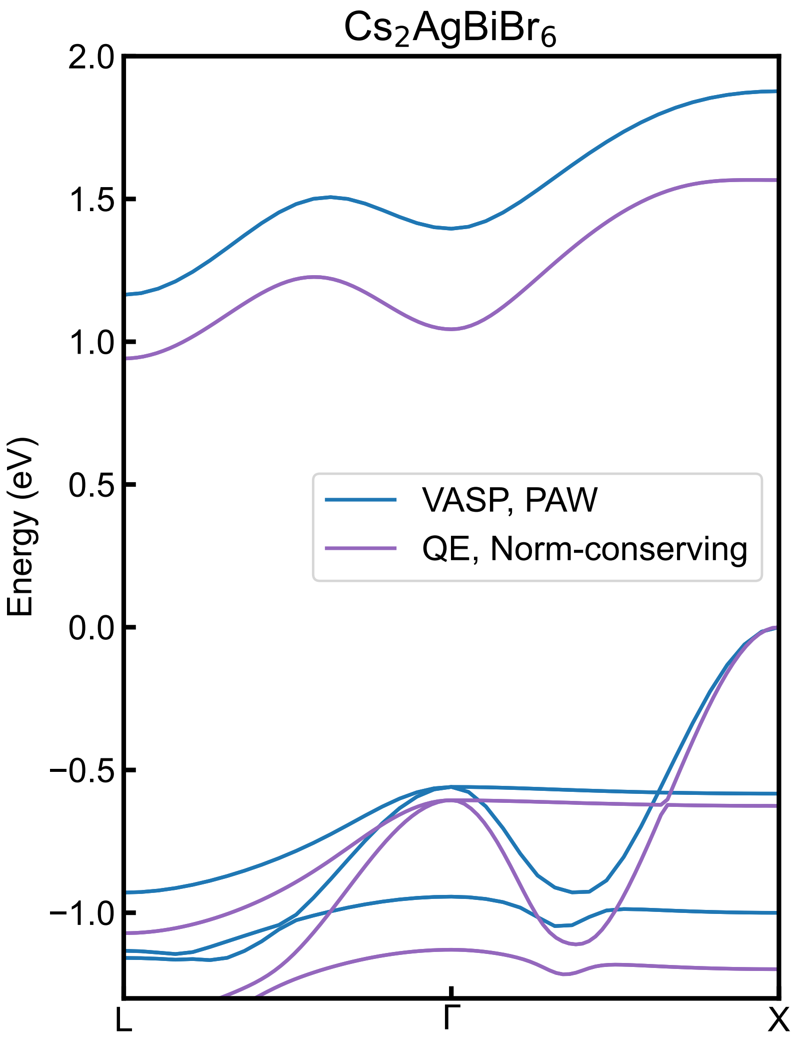

Figure S1: Bandstructure of $Cs_2AgBiBr_6$ as calculated with VASP (blue) and QUANTUM ESPRESSO (purple). In both bandstructures, the valence band maximum at X is the zero of the energy scale.

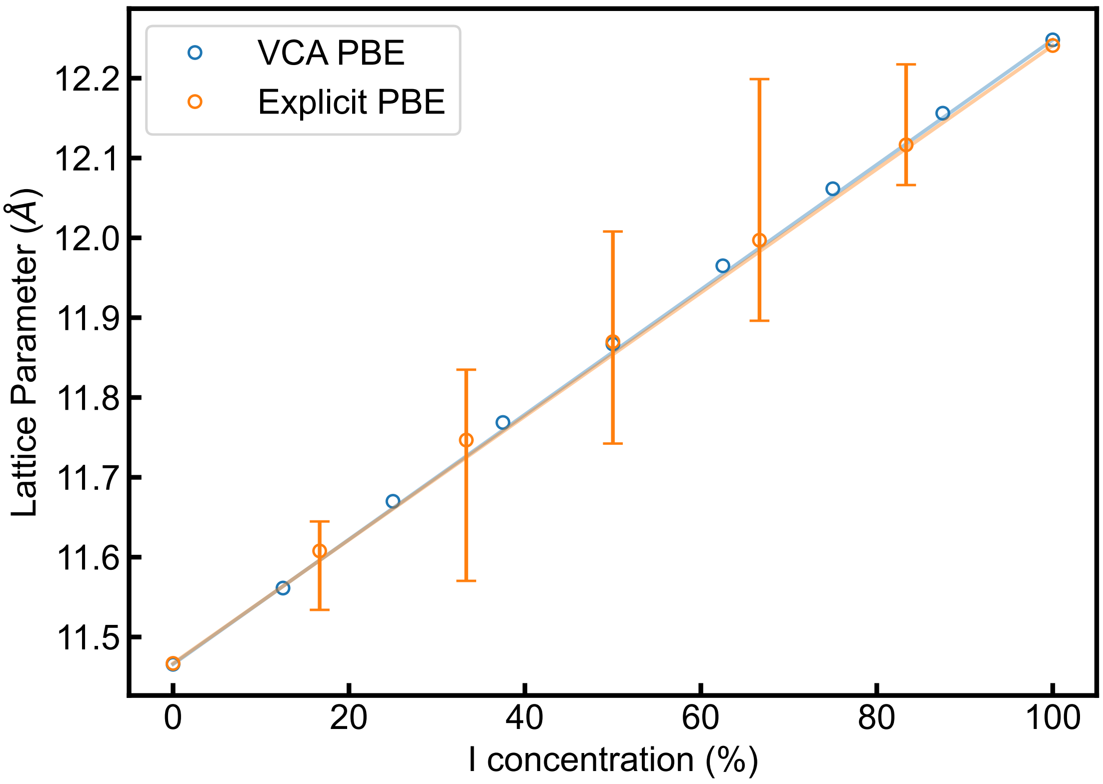

Figure S2: Lattice parameters as a function of I concentration using the VCA (blue dots) and an explicit alloy model of the structure (orange dots). Explicit structural models feature significant structural distortions and lattice vectors of different length. The orange dots correspond to an average over the lattice vector lengths. We use the error bars as a measure of the variation in lattice vector length, where the bottom (top) bar corresponds to the shortest (longest) lattice vector for each composition. Blue and orange lines show the expected linear relationship between the two endpoints.

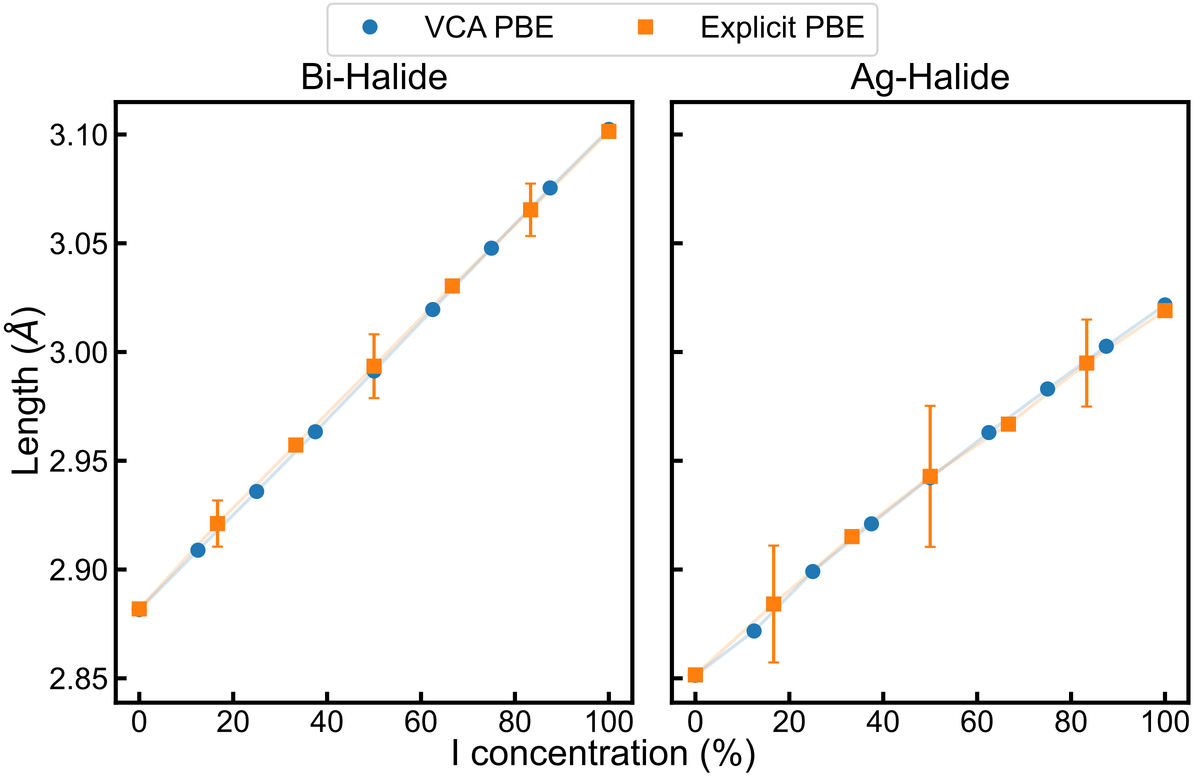

Figure S3: Average Bi-halide and Ag-halide bond lengths as a function of I concentration using the VCA (blue dots) and an explicit alloy model of the structure (orange squares). The orange squares correspond to averages and error bars are standard deviations. Blue and orange lines are guides to the eyes.

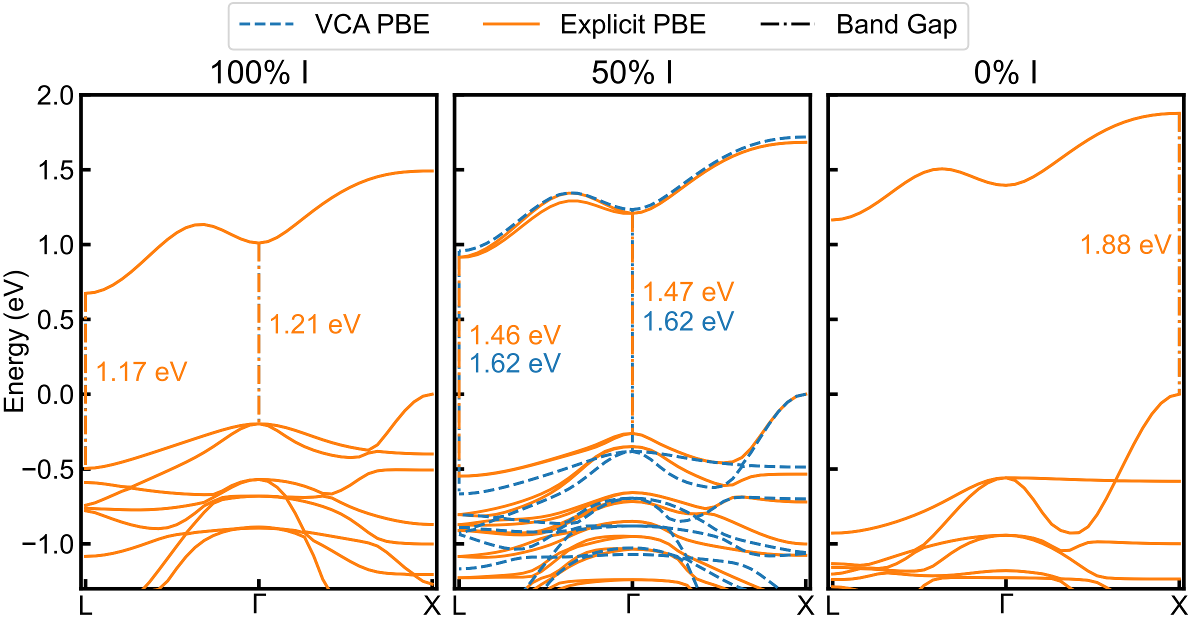

Figure S4: DFT-PBE+SOC bandstructures of Cs2AgBiI6, $Cs_2AgBiBr_{0.5}I_{0.5}$, and $Cs_2AgBiBr_6$ calculated with VASP. For the mixed composition, we are comparing the VCA with an explicit alloy model of the structure. Dashed vertical lines indicate the direct band gaps at Γ and L, which are nearly degenerate in our VASP calculations. Text indicates the band gap at the location of the dashed vertical line.

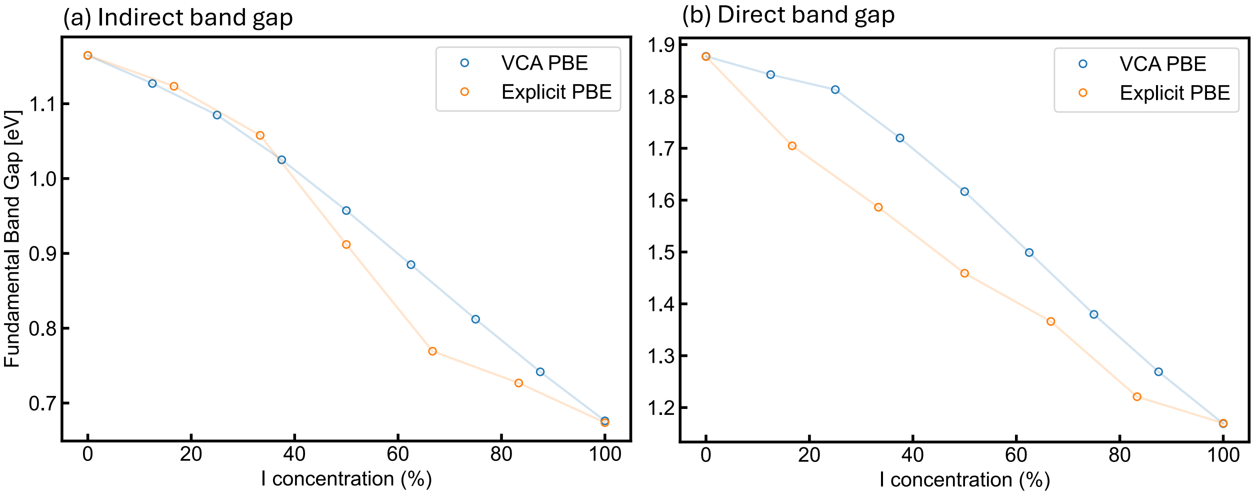

Figure S5: DFT-PBE+SOC (a) indirect and (b) direct bandgaps as a function of I concentration using the VCA (blue dots) and an explicit alloy model of the structure (orange dots). Orange and blue lines are guides to the eye.

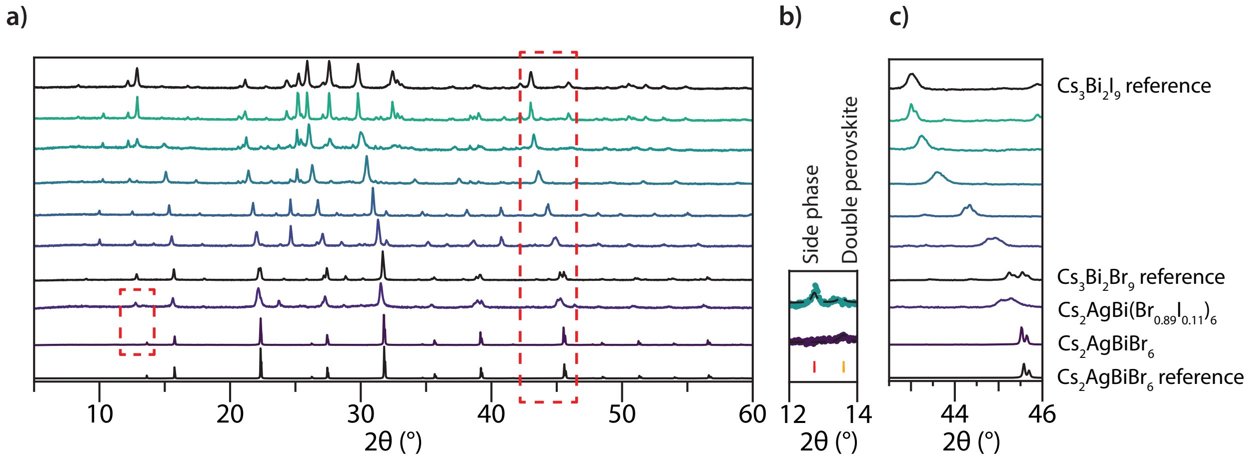

Figure S6: Experimental X-ray diffraction patterns of $Cs_2AgBi(Br_{1-x}I_x)_6$ and $Cs_3Bi_2(Br_{1-y}I_y)_9$ powders.

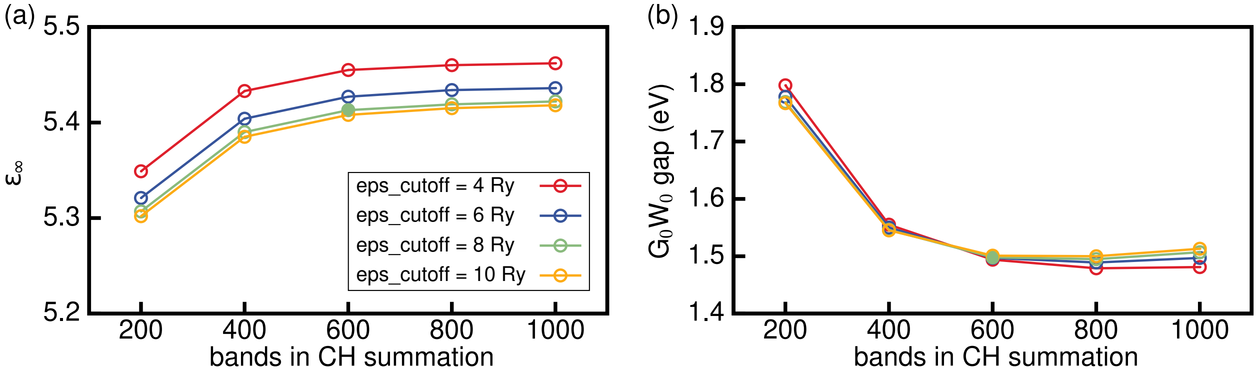

Figure S7: Convergence tests for the G_0W_0 parameters, for $Cs_2AgBiBr_6$: a) static dielectric constant ε_∞, b) QP band gap (indirect gap $X^{VBM} \rightarrow L^{CBM}$). The closed symbols show the parameters that ensure convergence (i.e. 8 Ry polarizability cutoff and 600 bands).

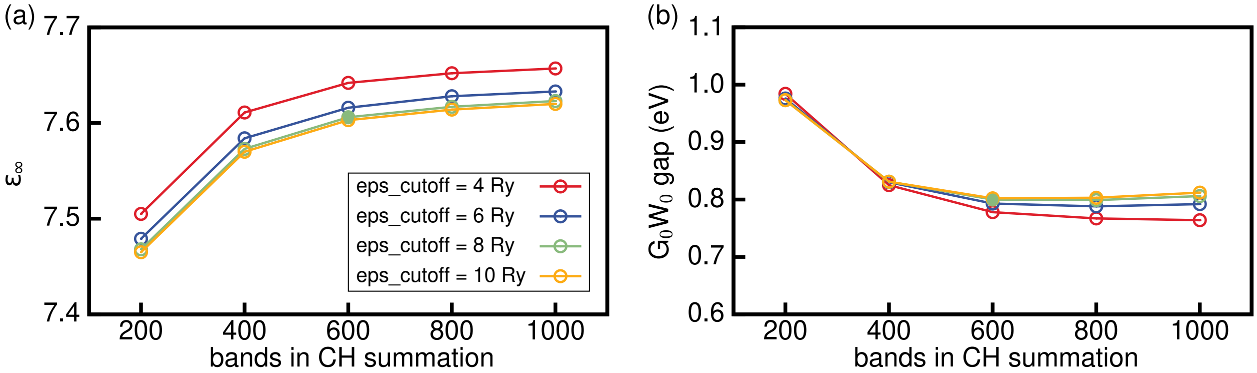

Figure S8: Convergence tests for the G_0W_0 parameters, for Cs_2AgBiI_6: a) static dielectric constant ε_∞, b) QP band gap (indirect gap $X^{VBM} \rightarrow L^{CBM}$). The closed symbols show the parameters that ensure convergence (i.e. 8 Ry polarizability cutoff and 600 bands).

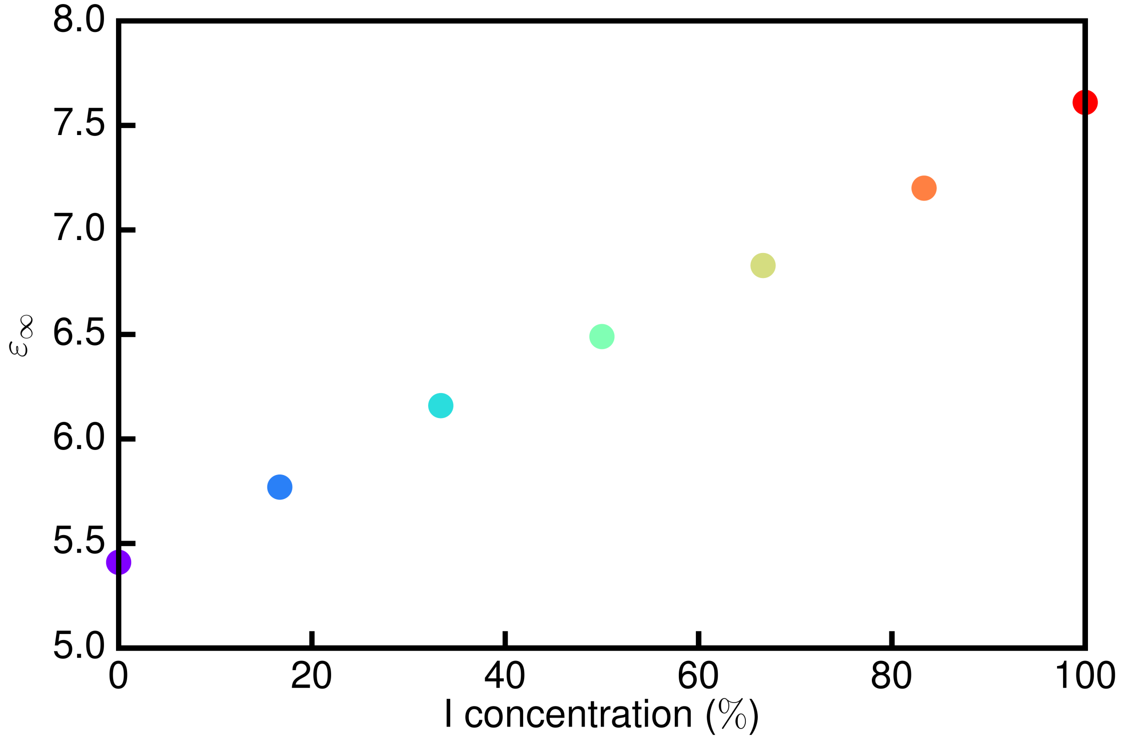

Figure S9: Static dielectric screening ε_∞ of $Cs_2AgBiI_xBr_{1-x}$, where x is the I concentration.

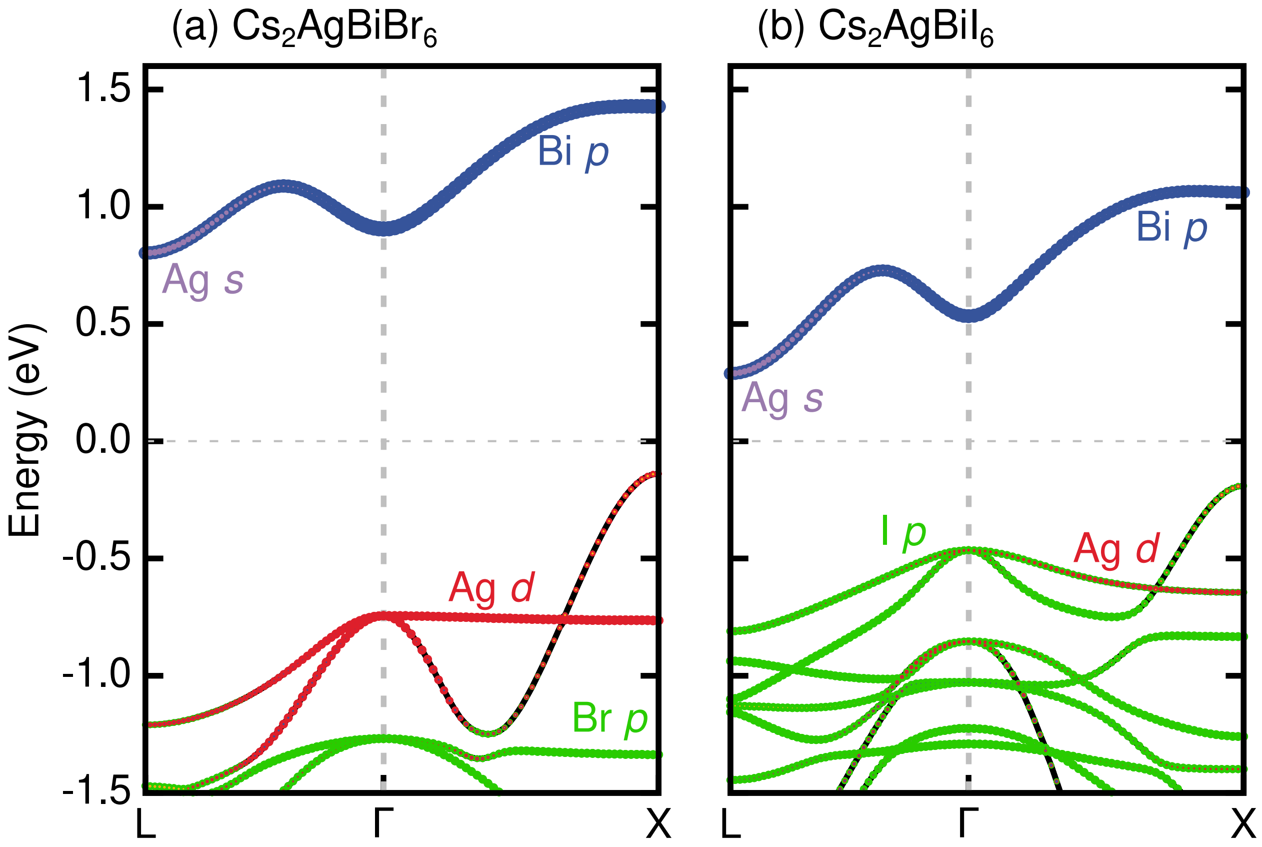

Figure S10: DFT-PBE electronic band structure of (a) $Cs_2AgBiBr_6$ and (b) Cs_2AgBiI_6, along the path L[1/2, 1/2, 1/2]$2\pi/a$ $\rightarrow$ Γ[0, 0, 0] $\rightarrow$ X[0, 1, 0]$2\pi/a$. The colored dots represent the orbital character of the bands and their size is proportional to the percentage contribution of the orbital character to the electronic bands. Cs-derived orbitals do not contribute to the states near the band edges.

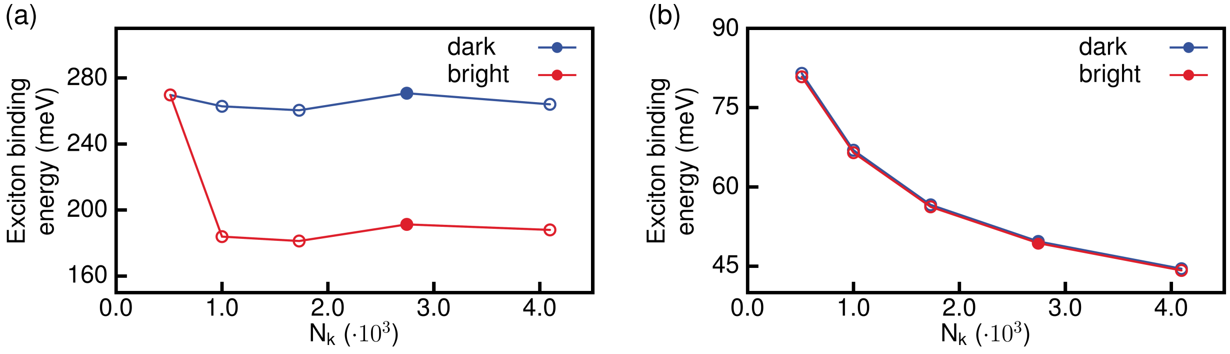

Figure S11: Convergence of exciton binding energy of the first dark (blue) and first bright (red) excited states for (a) $Cs_2AgBiBr_6$ and (b) Cs_2AgBiI_6. The closed symbols show the parameters that ensure convergence (i.e. a fine grid of $14 \times 14 \times 14$ **k**-points).

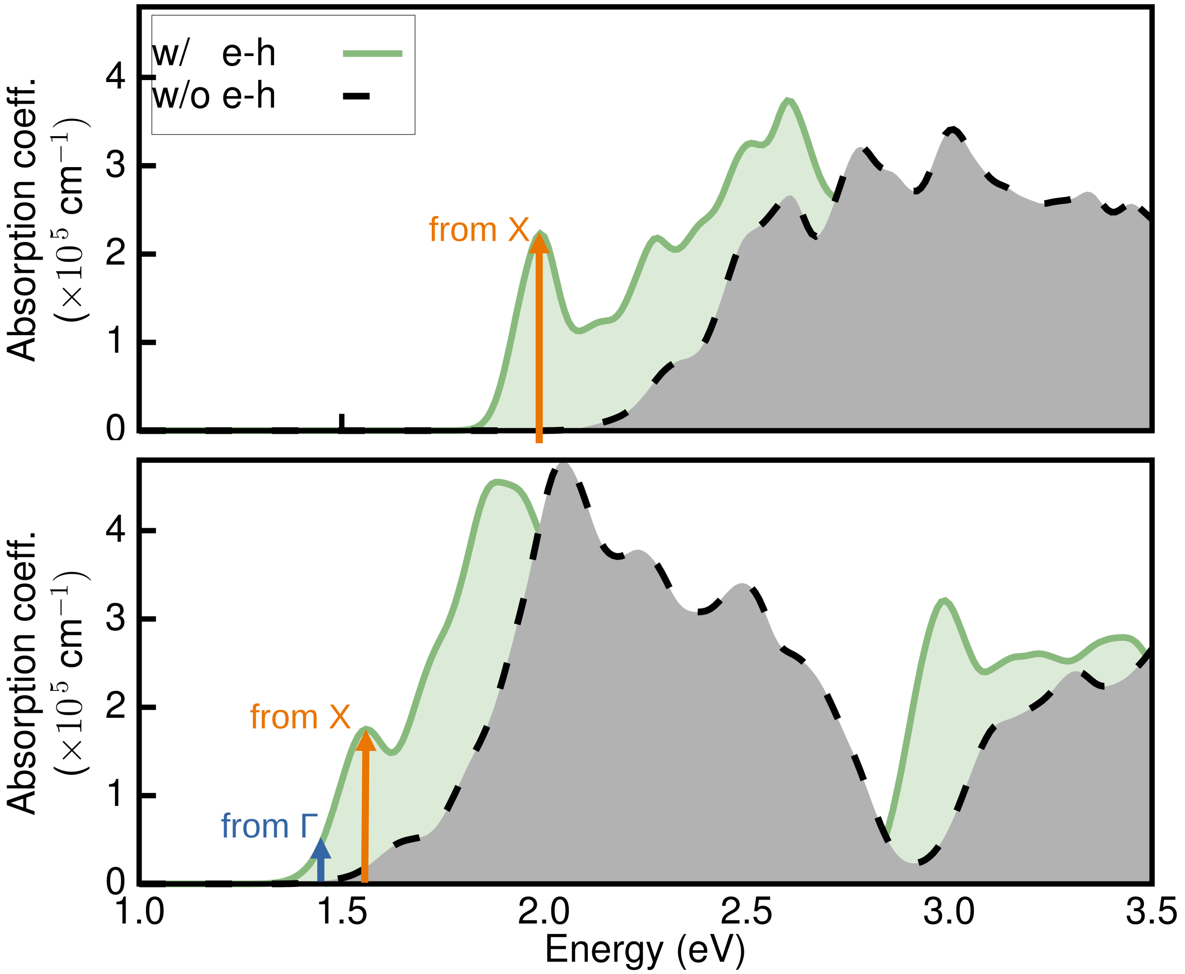

Figure S12: Linear optical absorption spectrum of (a) $Cs_2AgBiBr_6$ (top) and (b) Cs_2AgBiI_6 (bottom), calculated using the random phase approximation (RPA) (black) and the G_0W_0@PBE+BSE approach (green).

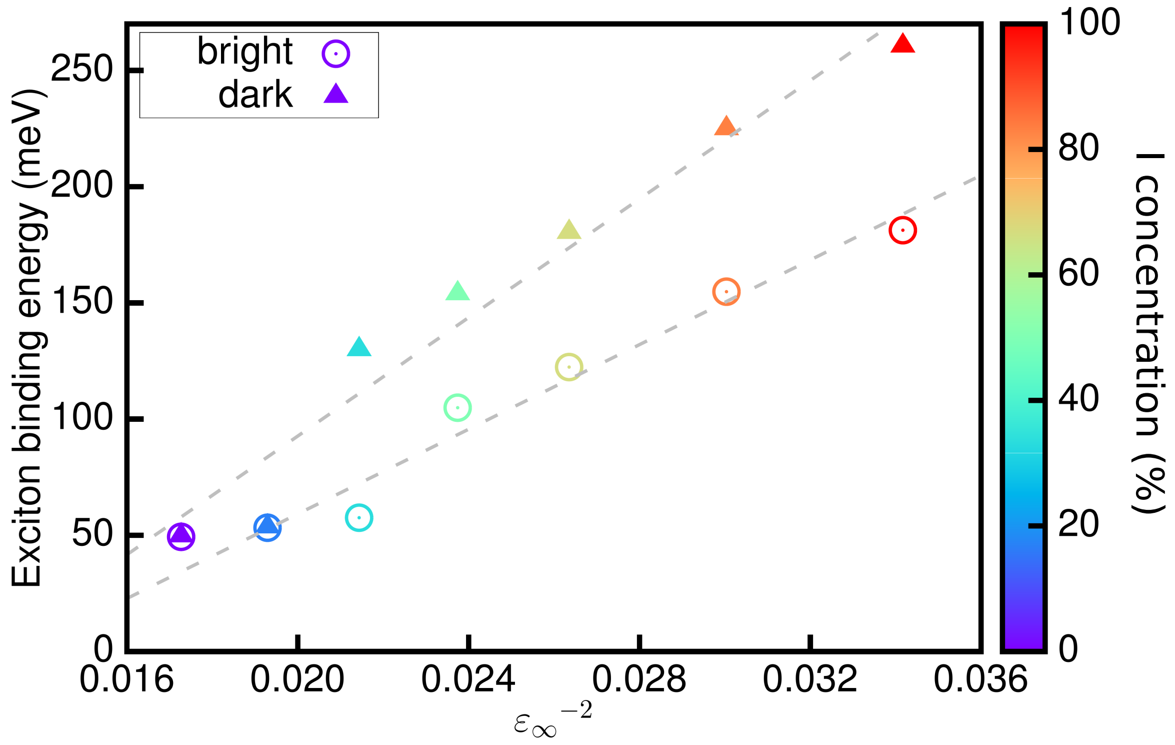

Figure S13: G_0W_0@PBE+BSE Exciton binding energy of first dark (triangles) and first bright (circles) transitions in $Cs_2AgBiBr_xI_{1-x}$.

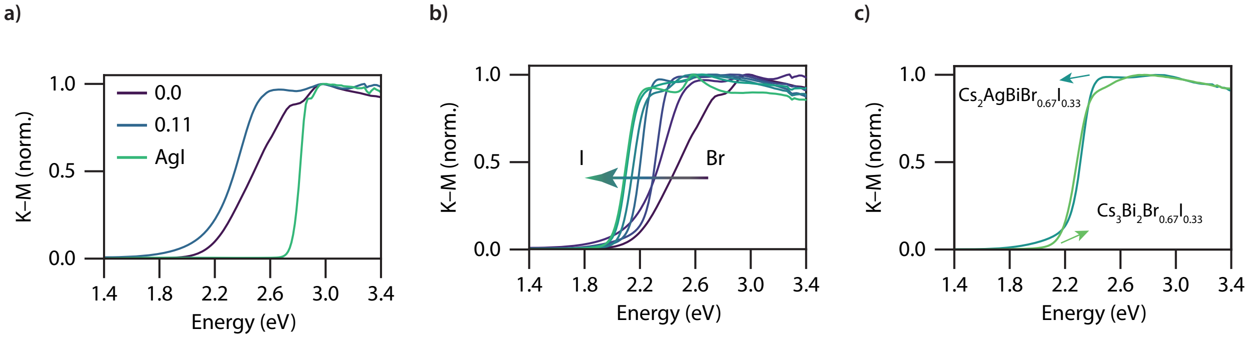

Figure S14: Experimental absorption spectra of $Cs_2AgBi(Br_{1-x}I_x)_6$ and $Cs_3Bi_2(Br_{1-y}I_y)_9$ powders. a) Kubelka–Munk transform of $Cs_2AgBiBr_6$, $Cs_2AgBiBr_{0.89}I_{0.11}$ and AgI powders as a function of photon energy. b) Kubelka–Munk transform of $Cs_2AgBiBr_6$, $Cs_2AgBiBr_{0.89}I_{0.11}$ and $Cs_3Bi_2(Br_{1-y}I_y)_9$. c) Kubelka–Munk transform of $Cs_2AgBi(Br_{0.67}I_{0.33})_6$ and $Cs_3Bi_2(Bi_{0.67}I_{0.33})_9$.

References

(1) Kresse, G.; Furthmüller, J. Efficient Iterative Schemes for Ab Initio Total-Energy Calculations Using a Plane-Wave Basis Set. *Phys. Rev. B* **1996**, *54*, 11169–11186.

(2) Blöchl, P. E. Projector Augmented Wave Method. *Phys. Rev. B* **1994**, *50*, 17953–17979.

(3) Filip, M. R.; Giustino, F. GW Quasiparticle Band Gap of the Hybrid Organic-Inorganic Perovskite CH3NH3PbI3: Effect of Spin-Orbit Interaction, Semicore Electrons, and Self-Consistency. *Phys. Rev B* **2014**, *90*, 245145.

(4) Scherpelz, P.; Govoni, M.; Hamada, I.; Galli, G. Implementation and Validation of Fully Relativistic GW Calculations: Spin-Orbit Coupling in Molecules, Nanocrystals, and Solids. *J. Chem. Theor. Comp.* **2016**, *12*, 3523–3544.

(5) Leppert, L.; Rangel, T.; Neaton, J. B. Towards Predictive Band Gaps for Halide Perovskites: Lessons from One-Shot and Eigenvalue Self-Consistent GW. *Phys Rev Mater.* **2019**, *3*, 103803.

(6) Sharifzadeh, S.; Darancet, P.; Kronik, L.; Neaton, J. B. Low-Energy Charge-Transfer Excitons in Organic Solids from First-Principles: The Case of Pentacene. *J. Phys. Chem. Lett.* **2013**, *4*, 2197.

(7) Biega, R.-I.; Filip, M. R.; Leppert, L.; Neaton, J. B. Chemically Localized Resonant Excitons in Silver–Pnictogen Halide Double Perovskites. *The Journal of Physical Chemistry Letters* **2021**, *12*, 2057–2063.

(8) Wannier, G. H. The Structure of Electronic Excitation Levels in Insulating Crystals. *Phys. Rev.* **1937**, *52*, 191–197.

(9) Schindlmayr, A. Excitons with Anisotropic Effective Mass. *Euro. J. Phys.* **1997**, *18*, 374–376.
